# How endothelial cell metabolism shapes blood–brain barrier integrity in neurodegeneration

**DOI:** 10.3389/fnmol.2025.1623321

**Published:** 2025-06-25

**Authors:** Jiaxin Wang, Yuchun Chen, Shiteng Chen, Zihan Mu, Jun Chen

**Affiliations:** ^1^School of Pharmacy, Shandong University of Traditional Chinese Medicine, Jinan, China; ^2^School of Traditional Chinese Medicine, Shandong University of Traditional Chinese Medicine, Jinan, China; ^3^School of Acupuncture-Moxibustion and Tuina, Shandong University of Traditional Chinese Medicine, Jinan, China

**Keywords:** endothelial cell, metabolism, blood–brain barrier, stroke, Alzheimer’s disease, multiple sclerosis, aging

## Abstract

**Background/objective:**

Endothelial cells, a monolayer of cells adjacent to blood vessels, play a critical role in maintaining vascular function through metabolic pathways such as glycolysis, fatty acid, and amino acid metabolism. Recent studies have revealed their significant involvement in neurodegenerative diseases, although the underlying mechanisms remain unclear.

**Methods:**

By reviewing literature from the past decade, we summarized the metabolic alterations and functional changes of endothelial cells in neurological disorders.

**Results:**

In neurodegenerative diseases such as stroke, Alzheimer’s disease, multiple sclerosis, and aging, metabolic dysregulation in cerebral vascular endothelial cells disrupts their normal function and is closely associated with blood–brain barrier impairment.

**Conclusion:**

Aberrant endothelial cell metabolism compromises the integrity of the blood–brain barrier and exacerbates the pathological progression of neurodegenerative diseases. Our review further explores the therapeutic potential of targeting endothelial cell metabolism in various pathological contexts, aiming to provide novel insights for the prevention and treatment of related disorders.

## 1 Introduction

The term “metabolism” originates from the ancient Greek word μεταβολή (metabolē), which entered scientific nomenclature in the late 19th century to describe “the totality of biochemical transformations through which cellular components in the cytosol and nucleus undergo renewal, modification, or preparation for excretion” (Li et al., 2019). This complex network encompasses over 4,000 enzymatic reactions that fulfill diverse biological functions, including nutrient conversion into biomass, energy production, redox balance maintenance, and intermolecular transformations essential for cellular homeostasis (Li et al., 2019). Endothelial cells (ECs), constituting the body’s most metabolically dynamic monolayer of cells. During quiescence, these cells maintain thromboresistance, anti-inflammatory properties, vascular tone regulation, and barrier integrity. Upon exposure to stress stimuli, ECs undergo metabolic reprogramming to meet heightened energy demands during their transition to an activated state. This adaptive process coordinates multiple vascular functions through: lipoprotein transport modulation (Kang et al., 2024), angiogenesis (Potente et al., 2011; Eelen et al., 2018; Eelen et al., 2015), vascular permeability (Phoenix et al., 2022), inflammatory response (Tanaka et al., 2024), leukocyte adhesion/extravasation (Hordijk, 2016), redox (Santoro, 2018) and other aspects to ensure vascular homeostasis and health. The recognition of EC-specific metabolic pathways in maintaining vascular homeostasis has recently garnered significant scientific attention.

The blood–brain barrier (BBB), a specialized component of the neurovascular unit, constitutes a unique cytoarchitectural complex in the central nervous system. This dynamic interface comprises cerebral capillary ECs interconnected with pericytes, astrocyte end-feet, and the basement membrane, forming a coordinated multicellular system (Abbott et al., 2010). Functionally, the BBB demonstrates selective molecular filtration capacity, effectively restricting blood-borne neurotoxic substances while maintaining cerebral homeostasis through precise regulation of the brain’s chemical and cellular microenvironment (Abbott et al., 2010). Central nervous system ECs exhibit four distinctive barrier-enhancing characteristics: (1) Continuous tight junctions (TJs) complexes that eliminate paracellular permeability, (2) Expression of specialized transport systems governing bidirectional substrate flux, (3) Extremely low rates of transcellular vesicle trafficking, termed transcytosis, to limit transcellular transport through the vessel wall, and (4) Constitutively low expression of leukocyte adhesion molecules, establishing immunological quiescence at the cerebrovascular interface (Langen et al., 2019). Pathological analyses reveal that BBB dysfunction manifests across neurological disorders including stroke, Alzheimer’s disease (AD), multiple sclerosis (MS), and aging processes. These conditions correlate with TJs structural disintegration, metabolic dysregulation involving glucose, fatty acid, and amino acid pathways, and consequent barrier impairment in cerebral ECs. This review screened the relevant literature on metabolic changes of ECs and BBB destruction in neurodegenerative diseases in the past decade, and systematically investigated the metabolic characteristics of brain ECs under physiological conditions and their pathological alterations during BBB destruction, aiming at elucidating the mechanism and providing novel therapeutic strategies for neurodegenerative diseases.

### 1.1 Glucose metabolism

There are two main metabolic pathways in ECs, glycolysis and oxidative phosphorylation, which play a role in anaerobic and aerobic conditions, respectively, and their main process of converting glucose to pyruvate for the rapid generation of adenosine triphosphate (ATP) is via the glycolytic pathway (De Bock et al., 2013b). ECs are heavily dependent on glucose, but have lower levels of oxidative phosphorylation and less mitochondrial content than other types of oxidized cells (De Bock et al., 2013b). On the other hand, although oxidative phosphorylation of glucose produces up to 36 ATP molecules, and per glucose molecule produces a net total of only 2 ATP molecules in glycolysis, at first sight, it might seem enigmatic why quiescent ECs do not take full advantage of their easy access to oxygen. This might be a mechanism to protect ECs from oxidative damage by keeping reactive oxygen species (ROS) levels in check, and is also the reason that preferentially utilizes glycolysis over oxidative metabolism (De Bock et al., 2013a). In addition, filopodia of tip cells explore and extend into hypoxic tissues, away from perfused blood vessels, where oxygen levels drop faster than glucose levels, making oxidative metabolic trouble. Also, glycolysis produces ATP with faster kinetics, necessary for the rapid revascularization of hypoxic tissues before their demise (De Bock et al., 2013b). Most of the pyruvate produced by glycolysis is eventually converted to lactic acid by lactate dehydrogenase; hexokinase is the first rate-limiting enzyme in the glycolysis process, which can catalyze the conversion of glucose to glucose-6-phosphate; phosphofructokinase 1 is the second rate-limiting enzyme that converts fructose-6-phosphate to fructose 1,6-bisphosphate and adenosine diphosphate (ADP); pyruvate kinase is the third rate-limiting enzyme which converts phosphoenolpyruvate to pyruvate and generates ATP, and multiple rate-limiting enzymes work together to control the rate of glucose metabolism (De Bock et al., 2013b; Leung and Shi, 2022). Furthermore, the Crabtree effect has been found in human umbilical vein ECs, and high glucose levels inhibit carbohydrate or glutamine-driven mitochondrial respiration, possibly increasing the oxidation of fatty acids in certain cells (Krützfeldt et al., 1990; Dagher et al., 2001; Koziel et al., 2012).

**Figure 1:**
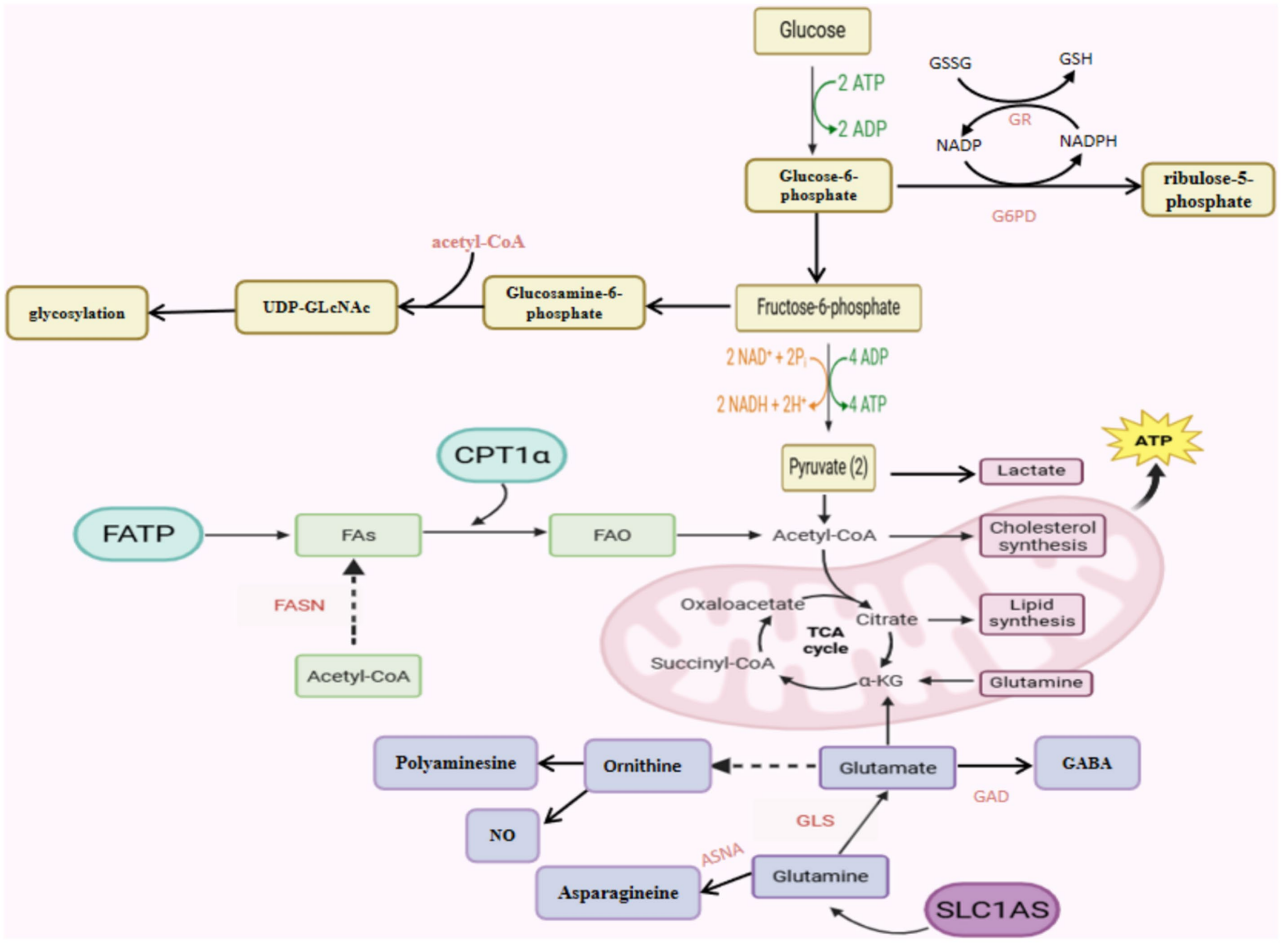
Schematic representation of the metabolic pathways of an endothelial cell controlling its function.

Endothelial function is also affected by other branched metabolic pathways of glycolysis (Goveia et al., 2014). For example, the polyol pathway, which depletes coenzyme II (NADPH), may reduce the formation of the ROS reducing agent glutathione, thus increasing oxidative stress in ECs, and the polyol pathway can metabolize up to 33% glucose when hexokinase is saturated in hyperglycemia (Clyne, 2021). The pentose phosphate pathway (PPP) occurs in the cytoplasm of most organisms which is a collateral branch of glycolysis, and glucose intermediates provide fuel for PPP that can convert glucose-6-phosphoribose to ribose 5-phosphate, whose activity can promote the production of NADPH and have an important impact on oxidative stress in ECs (Fuentes-Lemus et al., 2023; Stincone et al., 2015). ECs can also store large amounts of glucose in glycogen, which is mobilized in the event of glucose deprivation, and if the rate-limiting enzyme in glycogen degradation (glycogen phosphorylase) is inhibited, ECs migration and viability are impaired (Vizán et al., 2009). Several findings suggest that high glucose upregulates the protein level of Hypoxia-inducible factor 1 alpha (HIF-1α), and increases the transcriptional activity of hypoxia-inducible factor 1 (HIF-1) in ECs. As VEGF is an essential downstream effector of HIF-1, glucose-induced VEGF overexpression in ECs depends on HIF-1 activation. VEGF inhibition improves occludin and zonula occludens-1 (ZO-1) expression patterns, thereby attenuating endothelial leakage and BBB disruption (Yan et al., 2012). In the case of hypoglycemia we find that prolonged, severe hypoglycemia with hypothermia caused a profound BBB dysfunction whereas normothermic hypoglycemia resulted in few cases of any noticeable increase in BBB permeability (Oztaş et al., 1985). In short, these reactions highlight the important role that glucose metabolism may play in antioxidant and maintenance of vascular ECs permeability (Figure 1).

### 1.2 Fatty acid metabolism

Fatty acid metabolism involves multiple processes, including fatty acid intake, storage, transport, oxidation, and fatty acid synthesis, although it is considered that only a small fraction of the ATP produced in ECs metabolism is produced by fatty acid metabolism (about 5%). Fatty acids focus on supporting other key functions such as nucleotide synthesis, post-translational protein modification, signal transduction pathway activation, and gene regulation (Yang et al., 2016; Liu and Dai, 2022). ECs exist fatty acid synthases (FASNs) required for fatty acid synthesis, which silencing reduces palmitoylation and subsequent membrane localization of nitric oxide synthase (NOS). Carnitine palmitoyltransferase 1a (CPT1a) is an important rate-controlling enzyme during fatty acid oxidation (Schoors et al., 2015; Harjes et al., 2016), which is responsible for the introduction of fatty acids into the mitochondria and the restriction of fatty acid flux (Weber et al., 2024). Pharmacological or genetic deletion of CPT1a leads ECs proliferation and germination defects *in vitro* and vivo without affecting ECs migration. In ECs, fatty acids are metabolized to acetyl-CoA, which helps maintain the tricarboxylic acid cycle (TCA) cycle and deoxy-ribonucleoside triphosphate (dNTP) synthesis. Supplementation the CPT1a depleted cells with acetate, a precursor to acetyl-CoA, to promote the TCA cycle, which restores deoxyribonucleoside triphosphate levels and rescues vascular germination defects (Schoors et al., 2015). Fatty acid oxidation is an essential process for energy generation and biomass synthesis, and is also essential for maintaining redox homeostasis.

Lipids are a major part of brain cell membranes, and fatty acids are one of the main components of lipids, including two key fatty acids, arachidonic acid and docosahexaenoic acid (DHA), which play important roles in neuroprotection, brain development, synaptogenesis, and neural differentiation (Sambra et al., 2021). As one of the important microbial metabolites, short chain fatty acids (SCFA) are essential for maintaining the integrity of BBB under different pathological conditions (Fock and Parnova, 2023). The mechanism of SCFA-induced improvement in BBB integrity appears to be based on more than just the restoration of TJs proteins. Given that oxidative stress and inflammation are the major causes of junctional complex disruption and barrier leakage, the ability of SCFAs to inhibit pro-inflammatory nuclear factor kappa-B (NF-κB) and activate antioxidant Nuclear Factor erythroid 2-Related Factor 2 (Nrf2) pathways may explain their protective properties in a variety of barrier tissues (Fock and Parnova, 2023). MFSD2A is a sodium-dependent lysophosphatidylcholine (LPC) isotransporter protein, existed in the ECs that make up BBB, which uptakes DHA into the brain in the form of LPC (Nguyen et al., 2014). Furthermore, lipids transported by Mfsd2a establish a unique lipid environment that inhibits caveolae vesicle formation in CNS ECs to suppress transcytosis and ensure BBB integrity (Andreone et al., 2017). Claudin-5 is a major cell adhesion molecule that forms a paracellular barrier between ECs, induced an increase in BBB permeability with the absence of claudin-5 (Menard et al., 2017; Cheng et al., 2018; Lee et al., 2018), palmitoylation is achieved by linking reversible covalent palmitic acid molecules to cysteine residues on proteins, which is particularly important for the critical claudin-5. Palmitoylated claudin-5 is transferred to cholesterol-rich lipid rafts, where it dimerizes and limits cellular permeability to enhance brain endothelial barrier function (Rajagopal et al., 2019).

### 1.3 Amino acid metabolism

Glutamine is the most consumed amino acid in ECs, and its deprivation severely impairs EC proliferation and vascular germination. Glutamine depletion decreases glutathione levels, making ECs susceptible to ROS damage (Huang et al., 2017; Ju et al., 2021), and degrading TJs, ultimately increasing BBB permeability (Yang et al., 2021). When glutamine is depleted or catabolism is inhibited, ATP levels in ECs are significantly reduced. In ECs, 30% of the TCA carbon is derived from glutamine, which is comparable to the carbon derived from glycolysis and fatty acid metabolism (Nguyen et al., 2014). Glutamine is metabolized by glutaminase (GLS) to produce glutamate, which is both a metabolic intermediate of TCA and an important neurotransmitter in the brain, and mitochondria convert glutamate to *α*-ketoglutarate for ATP production, NADPH production, and fatty acid synthesis (Kim et al., 2017). Arginine is a semi-essential amino acid from glutamic acid as a substrate catalyzed by a variety of enzymes, and is involved in many cellular metabolic and signaling pathways of many cells through its different products of catabolism. In fact, arginine is not only involved in protein synthesis, but also a single substrate for endothelial NOS to produce an important vasoprotective molecule nitric oxide (NO) (Morris, 2009). Under normal conditions, NOS catalyzes the conversion of arginine and O₂ to NO and citrulline, utilizing electrons from NADPH and requiring tetrahydrobiopterin (BH_4_) and other cofactors. Reduced NO bioavailability or NO deficiency is most common attribution to decreased endothelial NOS activity in endothelial dysfunction. This deficiency promotes a vasospastic, prothrombotic, and inflammatory state in the vascular wall, which aligns with key Cerebral Small Vessel Disease mechanisms: hypoxia/ischemia and increased BBB permeability (Dobrynina et al., 2023). The combination of asparagine and α-ketoglutarate alleviate ECs deficiency caused by glutamine deprivation, suggesting that asparagine plays a key role in the response of ECs to glutamine deprivation. Silencing asparagine synthetase (ASNS) impairs ECs proliferation, illustrating the importance of asparagine for vascular sprouting (Huang et al., 2017).

Methionine and cysteine are sulfur-containing amino acids, which are highly oxidizing and make both amino acids extremely sensitive to many forms of reactive oxygen species and therefore critical to controlling the conduct of cellular redox reactions (Bin et al., 2017). Methionine participates in various metabolic processes as a major methyl donor and a precursor to sulfur compounds. Cysteine is catabolized to form important redox cofactors such as glutathione and hydrogen sulfide, which occupy the active sites of several enzymes and can protect cells from oxidative stress by regulating the redox reaction of sulfhydryl residues. In summary, both methionine and cysteine metabolism in ECs affect oxidative stress through the involvement of metabolic intermediates (Carter and Morton, 2016; Oberkersch and Santoro, 2019). In addition, homocysteine (Hcy) increased cytokine levels in the brain suggesting that inflammation might also be associated with the neuronal dysfunction observed in hyperhomocystinuric patients. Also, it is important to note that neuro-inflammation is often involved in the dysfunction of the BBB (Kamat et al., 2013).

## 2 Abnormal metabolism of ECs leads to damage to the BBB

ECs metabolic activity exerts tripartite regulatory control over BBB integrity through: modulation of paracellular permeability via TJs dynamics, maintenance of connexin-based gap junction networks, and structural integration with adjacent neurovascular unit constituents. Preservation of cerebral EC metabolic homeostasis is therefore paramount for sustaining BBB functional competence and intercellular crosstalk within the neurovascular microenvironment. To elucidate these mechanisms, we present four pathophysiological paradigms characterized by BBB dysfunction: stroke, AD, MS, and aging. This conceptual framework advances our understanding of metabolic regulation in BBB pathophysiology while identifying actionable targets for treating barrier-related neurodegenerative disorders (Table 1).

**Table 1:** List of neurological disorders diseases associated with endothelial cell (EC) metabolic deregulation.

Disease	Metabolic perturbation	Consequences
Stroke	ROS (Végran et al., 2011)↑, lactic acid (Song et al., 2024)↑, adenosine (Bynoe et al., 2015)↑	Promotes angiogenesis (Végran et al., 2011), Anaerobic glycolysis accelerates (Song et al., 2024), Formation of ischemic penumbra (Song et al., 2024)
Alzheimer’s disease	GLUT-1 (76)↓, Pyruvate dehydrogenase complex (Bubber et al., 2005)↓, KGDHC (Liu et al., 2004)↓	Aβ accumulation (Bubber et al., 2005), cognitive impairment (Bubber et al., 2005)
Multiple Sclerosis	Occluding (Stover et al., 1997)↓, VE-cadherin (Stover et al., 1997)↓, ICAM-1 (100)↑, P-gp (Stover et al., 1997)↑, GLUT-1 (100)↓	Immune cell extravasation (Stover et al., 1997), BBB permeability changes (Macor et al., 1991), inflammation (Macor et al., 1991)
Aging	NO (Stabenow et al., 2022)↓, PDHK (Hernanz et al., 2000)↓, Arg2 (Hernanz et al., 2000)↑, ROS (Hernanz et al., 2000)↑, L-aspartic acid (Ya et al., 2024)↓, L-glutamic acid (Ya et al., 2024)↓, L-Proline (Ya et al., 2024)↓, sn-glycero-3-phosphocholine (Ya et al., 2024)↓	Abnormal vascular morphology (Andjelkovic et al., 2023), Secretion of pro-inflammatory cytokines (Stabenow et al., 2022), Levels of oxidative stress rise (Ocaña et al., 2023), BBB integrity is broken (Ocaña et al., 2023)

GLUT-1, glucose transporter 1; NO, nitric oxide; ROS, reactive oxygen species; KGDHC, α-ketoglutarate dehydrogenase complex; ICAM-1, intercellular cell adhesion molecule-1; P-gp, P-glycoprotein; PDHK, Pyruvate dehydrogenase; LDHA, lactate dehydrogenase A; Arg2, Arginase2.

### 2.1 Stroke

Stroke is currently a major disease affecting human mortality, classified into two types: ischemic stroke and hemorrhagic stroke, with acute ischemic stroke accounting for 62.4% of the total incidence of strokes (Martin et al., 2024). One of the pathological markers of ischemic stroke is the breakdown of the BBB, which is characterized by changes in tight junction protein complexes, abnormalities in transport proteins and endocytotic transport mechanisms, and inflammatory damage, which trigger cognitive and motor impairments (Abdullahi et al., 2018). Ischemic stroke can lead to insufficient supply of oxygen and glucose, making it difficult to support the homeostasis of rat cerebral microvascular ECs, both Oxygen–glucose deprivation and simple medium exchange caused an increase in endothelial monolayer permeability. This correlated with reduced transcript levels of a number of TJs and tight junction-associated proteins (claudin-5, occludin, and ZO-1), as well as with altered transcript level of several transporters and receptors (Glucose transporter 1 (GLUT-1), Insulin Receptor (INSR), two members of the low density lipoprotein receptor family, Low-Density Lipoprotein Receptor (LDLR) and Low Density Lipoprotein Receptor-Related Protein 1 (LRP-1)) (Tornabene et al., 2019). And the hypoxic conditions lead to a sudden acceleration of anaerobic glycolysis and an increase in local lactate production, therefore Nicotinamide Adenine Dinucleotide (NAD) can only be regenerated through lactate, which is not only inefficient but may also induce cell apoptosis and contribute to the emergence of the ischemic penumbra (Song et al., 2024). Lactate also promotes angiogenesis by increasing the level of ROS in ECs, thereby stimulating NF-κB activity to influence barrier development and functional changes in BBB (Végran et al., 2011; Chen et al., 2020). Mitochondria are regarded as the “power station” of cells, and they play a key role in maintaining energy metabolism and determining cell vitality (Dong et al., 2019). Post-acute ischemic stroke hyperglycemia can aggravate the disruption of the BBB and reduce the overall energy metabolism level of cerebral microvascular ECs by inhibiting mitochondrial transfer (Xu et al., 2024). Some results show that both GLUT-1 and sodium-dependent glucose transporters (SGLT) still play a role at the BBB in the blood-to-brain transport of glucose during ischemic conditions, and inhibition of SGLT during stroke has the potential to improve stroke outcome. Pharmacological modulation of this novel BBB transporter could prove to be a brain vascular target in stroke (Vemula et al., 2009). N-methyl-d-aspartate receptor (NMDA-R) plays a crucial role in ischemic neuronal injury, cerebral vascular endothelial exposure to exogenous oxidative stress upregulates the expression of functional NMDA-R, and NF-κB activation is involved in the up-regulation of functional NMDA-R, treatment of bEnd3 cells with an inhibitor of NF-κB activation completely blocks ROS-induced up-regulation of NR1 subunit and depolarization of NMDA receptor after ROS exposure, Upregulation of NMDA-R expression increases endothelial response to glutamate stimulation and promotes BBB rupture (Neuhaus et al., 2011; Betzen et al., 2009).

Some studies have shown that ECs are more reactive and sensitive to hypoxia than pericytes (PCs) and astrocytes (ACs). HIF-1 is a master regulator of cellular adaptation to hypoxia, which translocates to the nuclei of brain capillary ECs cultured under hypoxic conditions and has been suggested as a potent therapeutic target in cerebral ischemia. The HIF-1 inhibition remarkably ameliorates ischemia-induced BBB disruption determined by Evans blue leakage (Yan et al., 2011; Ozgür et al., 2022; Engelhardt et al., 2015). Cluster of differentiation 39 (CD39) is an extracellular nucleoside triphosphate diphosphohydrolase expressed by ECs, and its primary function is to convert ATP/ADP into adenosine monophosphate (AMP), which is subsequently converted into adenosine by cluster of differentiation 73 (CD73) (Lee et al., 2021). Hypoxia, ischemia, and inflammation all stimulate local adenosine production and accumulation (Linden, 2001). Its receptor signaling is considered a key mediator by regulating tight junction proteins and maintaining the homeostasis of brain ECs function to affect BBB permeability (Bynoe et al., 2015; Jang and Song, 2024). Research has shown that treatment strategies targeting adenosine levels can reduce stroke-related brain damage and hypoxic–ischemic neuronal injury, restoring the normal functional levels of the BBB (Melani et al., 2012; Pedata et al., 2016). When a stroke occurs, due to increased paracellular and transcellular permeability along with endothelial injury, chemicals and fluids extravasate from the damaged BBB into the brain parenchyma resulting in vasogenic edema (Heo et al., 2005; Keaney and Campbell, 2015). The disruption of the BBB promotes progression of the damage and increases the risk of hemorrhage (Keep et al., 2014).

### 2.2 Alzheimer’s disease

AD is the most common form of dementia, characterized by typical clinical features, including amnestic memory impairment, language deterioration, and visuospatial deficits (Cummings, 2004). Impairment of the BBB in patients with AD is now a well-recognized pathology (Saraiva et al., 2016; Zlokovic, 2011; Chen et al., 2021), and previous disagreement about the involvement of the BBB in AD pathology may be due to the use of different assays, such as: the ratio of albumin in cerebrospinal fluid (CSF) and plasma (QAlb), soluble platelet-derived growth factor receptor *β* (sPDGFRβ), dynamic contrast-enhanced magnetic resonance imaging (DCE-MRI), and the use of a variety of other methods (Preis et al., 2024). In AD, there are specific changes in the cellular structure of BBB, alterations in the expression of amyloid β (Aβ) protein carriers, and abnormalities such as ECs apoptosis and metabolic damage (Kurz et al., 2022; Raut et al., 2021). Disruption of BBB integrity in AD was found to precede symptoms of cognitive impairment by several years. Damage to capillaries and BBB breakdown in the hippocampus and other brain regions are considered early biomarkers of cognitive dysfunction. This disruption results in nonselective entry of solutes from circulating blood into the extracellular fluid of the central nervous system, and leads to the perivascular accumulation of blood-derived fibrinogen, thrombin, albumin, and ferritin-containing deposits. These pathological changes further damage affected brain tissue and trigger neurodegeneration (Chen et al., 2023a; Alkhalifa et al., 2023). BBB breakdown in AD patients has been confirmed by autopsy studies that showed.

The pathological hallmarks of AD include glucose uptake reduced in the brain, the accumulation of Aβ and neurofibrillary tangles (Patrick, 2019). Compared to healthy controls, AD patients exhibit glucose metabolism is reduced in the brain’s ECs (Viña et al., 2021; Vogelsang et al., 2018), reflecting alterations between glycolysis and the mitochondrial TCA cycle (Austad et al., 2022). Animal studies have shown that GLUT-1 deficiency in brain ECs, rather than in astrocytes, due to BBB breakdown, which exacerbates AD, thereby accelerating Aβ accumulation and cognitive impairment (Winkler et al., 2015). Another possibility leading to the decrease in glucose is a key enzymes activity decrease in the TCA, with both pyruvate dehydrogenase complex, which provides acetyl-CoA, and alpha-ketoglutarate dehydrogenase complex (KGDHC) decreasing in AD (Bubber et al., 2005). Furthermore, a notable observation is that the reduction in pyruvate and fumarate, crucial intermediates of glycolysis and the TCA cycle, was consistently observed specifically in ECs across the three neurodegenerative conditions: Ischemic stroke, hemorrhagic stroke, and AD. This intriguing finding highlights the unique role of ECs metabolism in the pathogenesis of neurodegeneration (Guo et al., 2023). In AD, BBB INSR dysfunction leads to brain insulin resistance, linked to Aβ pathology (Leclerc et al., 2023). Advanced glycation end products (AGEs) accumulate in the AD brain, stimulating β-amyloid production and inducing tau hyperphosphorylation (Li et al., 2012). Azeliragon inhibits the receptor for advanced glycosylation end-products (RAGE) as a potential treatment to slow disease progression in patients with mild AD (Burstein et al., 2018). Under physiological conditions, Aβ binds to LRP-1 and is cleared from the brain via BBB transport mediated by P-glycoprotein (P-gp). Simultaneously, EC-expressed RAGE mediates peripheral Aβ reuptake. When BBB LRP-1 decreases and RAGE increases, Aβ clearance is impaired, causing cerebral accumulation. Both processes depend on mitochondrial homeostasis and ATP production (Chen et al., 2023a; Zou et al., 2020). We found that early tau-induced metabolic stress and increased glycolysis associated with pro-inflammatory EC activation, TJs loss, and BBB impairment (Guzmán-Hernández and Fossati, 2025). Reduced brain glucose uptake impairs the attachment of O-linked N-acetylglucosamine, leading to the hyperphosphorylation of tau protein and subsequent formation of neurofibrillary tangles, which accumulate in the microvascular system of the brain in AD patients (Liu et al., 2004), and the integrity of the BBB is maintained when tau expression is suppressed (Zhang et al., 2007; Zhang et al., 2021).

ApoE4 is associated with increased cognitive decline in aging, poor outcomes after stroke and traumatic brain injury, and is the main genetic risk factor for Alzheimer’s disease. ApoE4 brain ECs prefer oxidative phosphorylation to glycolysis, which leads to higher mitochondrial activity and production of reactive oxygen species and lower antioxidant levels (heme/bilirubin and glutathione). Higher levels of reactive oxygen species in apoE4 produce oxidative stress on proteins and lipids. At the same time, or due to higher mitochondrial activity, apoE4 is more inflammatory, which is characterized by chemokine production, immune cell adhesion and higher sensitivity of innate receptors to activation (Marottoli et al., 2021). It is found that the serum of exercise training individuals have a dimorphic effect on sirtuin 1 (SIRT-1) in apoE3 and apoE4 brain ECs and the serum after exercise training may send out the signal of SIRT-1 steady-state control, making them return to the general baseline (Weber et al., 2025).

### 2.3 Multiple sclerosis

MS is a complex heterogeneous disease characterized by inflammation, demyelination, and increased BBB permeability (Ruiz et al., 2019). One of the established markers is the presence of severe abnormalities in brain ECs, which lead to alterations in normal BBB function, allowing activated white blood cells to migrate across the endothelium into the central nervous system, where they drive inflammation and ultimately contribute to neurodegeneration (Macor et al., 1991). In the study of serum components in patients with relapsing–remitting multiple sclerosis, it was found that the patients impaired intercellular tightness by down-regulating occludin and VE-cadherin, resulting in changes to the permeability of BBB (Sheikh et al., 2020). Additionally, the up-regulation of intercellular adhesion molecules (ICAM-1) and P-gp facilitated the extravasation of immune cells, thereby affecting brain ECs (Sheikh et al., 2020). At the metabolic level, sera from patients with recurrent-remitting multiple sclerosis reduced the glycolytic activity of ECs, as measured by the extracellular acidification rate (ECAR) and oxygen consumption rate (OCR). This change is associated with downregulation of GLUT-1 expression and alterations in mitochondrial membrane potential (Sheikh et al., 2020). Furthermore, it was also found that their ECs released higher levels of ROS which indicated that the cells were in a pro-inflammatory state and also caused disruption of ICAM-1, ECs skeleton perturbation (stress fibers), and cytoskeletal signaling MSK1/2 and *β*-catenin phosphorylation (Sheikh et al., 2020).

The concentration of glutamate in the cerebrospinal fluid of patients with MS is increased, and its levels are correlated with the severity of the disease (Stover et al., 1997; Sarchielli et al., 2003). Research indicates that brain ECs (b. End3) produce ONOO− after exposure to glutamate, and it is noted that the generation of ONOO− in b. End3 cells stimulated by glutamate is mediated through NMDA receptor activation, with NO being produced through the up-regulation of specific NOS activity (Scott et al., 2007). Glutamate may mediate the breakdown of BBB in MS and Experimental Autoimmune Encephalomyelitis (EAE) through the action of ONOO−, and Studies on EAE suggest that pharmacological inhibition of specific glutamate receptors can suppress neurological symptoms and prevent BBB collapse (Scott et al., 2007).

### 2.4 Aging

In the aging process and age-related vascular lesions, cerebral capillaries exhibit morphological changes such as vascular tortuosity increased, circular twisting, irregular capillary diameters, and basement membranes thickened (Ek Olofsson and Englund, 2019). Clinical and experimental data indicate that the integrity of BBB is progressively compromised with advancing age, depending on two ongoing parallel processes: the damage to brain ECs, resulting in metabolic and structural changes (endothelium-centered processes), and the remodeling of the neurovascular unit (neurovascular unit-centered processes) (Andjelkovic et al., 2023). Here we focus on the effects of damage to brain ECs.

ECs aging is an indefinite state of cell cycle arrest with multiple biochemical and metabolic changes. Aging ECs are typically morphology flat and enlarged, increased polyploidy, decreased NO bioavailability, and secretion of multiple pro-inflammatory cytokines (Hwang et al., 2022). Indeed, we observed a global shift from ligand-specific receptor-Mediated Transcytosis (RMT) to non-specific caveolar transcytosis with age in brain ECs (Yang et al., 2020). Insulin is derived from islet β cells. It enters the central nervous system across BBB through a receptor-mediated, saturable process that is limited by the barrier system formed by tight junctions between ECs. Insulin resistance is a significant pathological phenomenon in age-related diseases, aging is related to the reduction of insulin and its receptor levels (Jiang et al., 2013; Sartorius et al., 2015; Akintola and van Heemst, 2015). As the main glucose transporter in the BBB, GLUT-1 is crucial for the uptake of glucose in the brain. The expression of GLUT-1 in ECs decreases slightly with age. The enhanced expression of GLUT-1 can alleviate the postoperative cognitive impairment in elderly mice (Chen et al., 2023b). Aging cells exhibit higher glycolytic activity and increased lactate levels, as well as TCA activity and mitochondrial respiration compared higher to young ECs. The pyruvate dehydrogenase complex (PDC) promotes oxidative glucose metabolism, and PDC catalyzes the irreversible decarboxylation of pyruvate to acetyl-CoA, which is inhibited by the action of Pyruvate dehydrogenase kinase (PDHK), which phosphorylates the E1-*α* subunit of Pyruvate Dehydrogenase (PDH) (Stabenow et al., 2022). Western blotting revealed that all four PDHK subtypes were down-regulated in senescent cells (inhibition of PDHK1, 2, 3, and 4 was approximately 50, 70, 30, and 40%, respectively), compared to younger cells (Stabenow et al., 2022). This is accompanied by a decrease in phosphorylation of PDHE1α at Ser293, indicating a higher activation of the PDC complex, which may be the reason for the increased TCA cycle activity in aging ECs. In addition to the reduced expression of PDHK, the expression of the lactate dehydrogenase-A (LDHA) subunit also significantly increased, which may mediate the observed increase in lactate production in aging ECs (Stabenow et al., 2022).

In the analysis of metabolites in brain ECs, four metabolites were found to be significantly lower in abundance compared to younger cells: L-aspartate (a precursor of several amino acids), L-glutamate (an important energy source under glucose deficiency), L-proline (a crucial component of collagen), and sn-glycerol-3-phosphocholine (a precursor for choline biosynthesis) (Hernanz et al., 2000). Homocysteine can accelerate the aging process, and plasma homocysteine levels in elderly subjects are significantly higher than in younger individuals, which can enhance oxidative effects on the endothelium (Ya et al., 2024). The abnormalities in several amino acid metabolites and elevated levels of oxidative stress leading to the loss of BBB integrity and impair its function. Arginase is an enzyme that metabolizes L-arginine into L-ornithine and urea, and is associated with endothelial dysfunction and aging, which have two isoforms, namely arginase 1 (Arg1) and arginase 2 (Arg2), with Arg2 being the predominant isoform induced in human ECs’ mitochondria (Yang and Ming, 2013; Yepuri et al., 2012; Jenkinson et al., 1996). Compared to non-senescent cells and young animals, aging ECs and aged mice exhibit an enhanced vulnerability in endothelial permeability and BBB dysfunction in response to hypoxia. Hypoxia increases the levels of arginase 2 (Arg2) in the brain vascular ECs of aged mice, and Arg2 levels elevated promote hypoxia-induced ROS production from the endothelium, leading to a reduction in TJs proteins in the hippocampal endothelium of hypoxic aged mice, consequently disrupting the BBB (Cheng et al., 2024). There is increasing evidence showing that mitochondrial oxidative stress plays a critical role in a range of age-related cellular impairments. According to the mitochondrial free radical theory of aging, the production of mitochondrial derived ROS and related mitochondrial dysfunction are the key driving forces in the process of aging (Tarantini et al., 2018).

## 3 Conclusion

This review synthesizes contemporary advances in ECs metabolic regulation and its critical role in maintaining BBB homeostasis. Emerging evidence positions EC metabolism as a promising therapeutic target for BBB-related pathologies, including ischemic stroke, AD, MS, and age-associated neurovascular dysfunction. Clinically approved agents such as dimethyl fumarate exemplify this therapeutic potential, demonstrating dual metabolic effects in cerebral ECs: enhanced glycolytic flux via phosphoglycerate dehydrogenase inhibition, and suppressed mitochondrial respiration through serine/glycine synthesis downregulation (Ocaña et al., 2023). These findings underscore the pathophysiological significance of glucose metabolism, lipid processing, and amino acid utilization in BBB maintenance.

However, current research on ECs metabolism predominantly focuses on neovascular ocular diseases, diabetes, and cancer, primarily investigating vascular complications arising from ECs dysfunction and pathological angiogenesis driven by metabolic abnormalities in ECs (Eelen et al., 2018). In contrast, studies on neurodegenerative diseases remain comparatively underdeveloped. Cerebral ECs exhibit distinct characteristics from their peripheral counterparts: high-resistance TJs that form robust intercellular connections, and limited transcytotic vesicles, collectively restricting both paracellular and transcellular molecular flux from the bloodstream into the brain parenchyma (Rubin and Staddon, 1999). Furthermore, cerebral ECs demonstrate elevated mitochondrial density compared to peripheral ECs, a feature that may confer enhanced metabolic flexibility and sustained cellular viability under glucose-deprived conditions (Weber et al., 2022). Although existing studies have preliminarily revealed fundamental differences in basic energy metabolism (such as glycolytic dominance versus mitochondrial respiratory efficiency) between peripheral ECs and brain ECs, significant knowledge gaps remain regarding their deeper metabolic regulatory networks. Key unresolved scientific questions include how targeting these metabolic nodes (e.g., inhibiting glycolytic kinases or enhancing fatty acid oxidation) could reverse BBB leakage in cerebrovascular diseases. Moreover, research on how metabolic crosstalk between ECs from different tissues (e.g., the impact of elevated circulating ketone bodies during peripheral inflammation on brain EC mitochondrial dynamics) dynamically regulates BBB tight junction protein degradation and transporter function remains fragmented. These knowledge gaps present dual challenges for cross-scale research: fundamentally, deciphering the molecular coupling mechanisms of the Metabolite-Epigenetics-BBB Axis, and translationally, developing spatiotemporally-specific metabolic intervention tools (e.g., BBB-penetrating nanocarriers loaded with metabolic reprogramming drugs). These areas represent both emerging research challenges and potential novel therapeutic strategies for BBB dysfunction.
